# Antioxidant Role of *PcGSTd1* in Fenpropathrin Resistant Population of the Citrus Red Mite, *Panonychus citri* (McGregor)

**DOI:** 10.3389/fphys.2018.00314

**Published:** 2018-03-29

**Authors:** Chong-Yu Liao, Ying-Cai Feng, Gang Li, Xiao-Min Shen, Shi-Huo Liu, Wei Dou, Jin-Jun Wang

**Affiliations:** ^1^Key Laboratory of Entomology and Pest Control Engineering, College of Plant Protection, Academy of Agricultural Sciences, Southwest University, Chongqing, China; ^2^Agricultural Genomics Institute at Shenzhen, Chinese Academy of Agricultural Sciences, Shenzhen, China

**Keywords:** *Panonychus citri*, fenpropathrin resistance, RNA-seq, glutathione *S*-transferases, antioxidant function

## Abstract

The citrus red mite, *Panonychus citri*, a major citrus pest distributed worldwide, has evolved severe resistance to various classes of chemical acaricides/insecticides including pyrethroids. It is well known that the resistance to pyrethroids is mainly caused by point mutations of voltage-gated sodium channel gene in a wide range of pests. However, increasing number of evidences support that pyrethroids resistance might also be resulted from the integrated mechanisms including metabolic mechanisms. In this study, firstly, comparative analysis of RNA-seq data showed that multiple detoxification genes, including a GSTs gene *PcGSTd1*, were up-regulated in a fenpropathrin-resistant population compared with the susceptible strain (SS). Quantitative real time-PCR results showed that the exposure of fenpropathrin had an induction effect on the transcription of *PcGSTd1* in a time-dependent manner. *In vitro* inhibition and metabolic assay of recombinant PcGSTd1 found that fenpropathrin might not be metabolized directly by this protein. However, its antioxidant role in alleviating the oxidative stress caused by fenpropathrin was demonstrated via the reversely genetic experiment. Our results provide a list of candidate genes which may contribute to a multiple metabolic mechanisms implicated in the evolution of fenpropathrin resistance in the field population of *P. citri*. Furthermore, during the detoxification process, *PcGSTd1* plays an antioxidant role by detoxifying lipid peroxidation products induced by fenpropathrin.

## Introduction

The citrus red mite, *Panonychus citri*, is one of main citrus pests with worldwide distribution (Gerson, 2003). Currently, the most effective way to prevent its population outbreak in field is always chemical acaricide spraying. However, as the long-term continual abuse of chemical synthesis compounds, along with its specific biological characterization such as high fecundity, short life cycle, and parthenogenesis, the citrus red mite has evolved severe resistance to various classes of chemical acaricides/insecticides including pyrethroids (Hu et al., 2010).

Pyrethroids, a class of synthetic insecticides and is modified from the structure of pyrethrins, have six insecticidal components of the natural insecticide pyrethrum (Elliott, 1995). The mode of action of pyrethroids on arthropods are known to alter their normal nerves function through affecting the permeability of voltage-gated sodium channels (Soderlund and Bloomquist, 1989). Attributed to their high efficiency and low mammalian toxicity, a mass of pyrethroids with analogous structure have been applied widely in agricultural, veterinary, medical, and household pest control. Nevertheless, widespread and indiscriminant application of pyrethroids have resulted in the emergence of resistance in various pests.

There are plenty of results found upon the past research providing the convincing evidence that the resistance to pyrethroids was mainly caused by single or multiple point mutations of voltage-gated sodium channel gene in a wide range of pests. For instance, two amino-acid substitutions of voltage-gated sodium channel in domain IIIS6 and the II/III intracellular interlinker were identified in a highly pyrethroids resistant strain of *Tetranychus urticae* (Tsagkarakou et al., 2009). The conserved point mutation of sodium channel (F1538I) has also been identified in a fenpropathrin resistant strain of *P. citri* (Ding et al., 2015). Even so, an increasing number of evidences support that pyrethroids resistance might also be resulted from the integrated mechanisms other than target site mutations. The elevated metabolic detoxification mediated by cytochrome P450-dependent monooxygenases, carboxylesterases (CarEs), and glutathione *S*-transferases (GSTs) have been associated as well with pyrethroids resistance in various pests. Two P450s, *CYP6P3*, and *CYP6M2*, were identified to be upregulated in three populations of *Anopheles gambiae* through microarray analysis using its detox chip (Djouaka et al., 2008). Both of enhanced oxidation by P450 and hydrolysis by esterase isozymes contribute to the fenvalerate resistance of *Helicoverpa armigera* (Wu et al., 2011). The transcripts of carboxylesterase showed over-expression in fenpropathrin resistant strains of *T. cinnabarinus* (Shi et al., 2016a) and *P. citri* (Shen et al., 2016). By the method of enzymatic analysis, elevated activities of GSTs were detected in pyrethroids resistant strains of *Tribolium castaneum* (Reidy et al., 1990), *Nilaparvata lugens* (Vontas et al., 2001), and *T. cinnabarinus* (He et al., 2009). Pyrethroids resistance in arthropods has also been associated with the upregulation of ATP-binding cassette transporters (ABC transporters), which have essential roles in transporting toxicants out of the cell (Bariami et al., 2012; Mamidala et al., 2012). The proposed mechanisms of pyrethroids resistance also involves the thickening of cuticle that may prevent or reduce pyrethroids penetration and absorption (Wood et al., 2010). Alternatively, increased level of antioxidants could have a protective mechanism against the oxidative stress induced by pyrethroids (Muller et al., 2007, 2008). It is notable that, the GSTs have also been implicated in reducing oxidative stress. For example, the elevated levels of GSTs present in a pyrethroids resistant strain alleviate the induced lipid peroxidation by pyrethroid in *N. lugens* (Vontas et al., 2001).

GSTs (EC 2.5.1.18), distributed widely in living organisms including insects and mites, are one major group of detoxification enzymes. They play dominant roles in the detoxification of both endogenous and xenobiotic compounds, and also involves in protection against oxidative stress (Enayati et al., 2005). The elevated GSTs activity and/or transcripts of individual GSTs gene have been related to the resistance not only to major class of insecticides (Vontas et al., 2001; Enayati et al., 2005; Lumjuan et al., 2005), but also to acaricides including fenpropathrin (He et al., 2009; Cong et al., 2016). Nevertheless, the concrete pathway in which pyrethroids was detoxified or metabolized to non-toxic compounds is obscure. Early study showed that GSTs play roles in conferring resistance by detoxifying lipid peroxidation products induced by pyrethroids (Vontas et al., 2001, 2002). Recently, recombinant GSTe2 of mosquitoes have been detected to be able to catalyze directly the metabolism of permethrin, although the corresponding metabolites remains to be identified (Riveron et al., 2014). In addition that, GSTs likely participate in pyrethroids detoxification in insect through sequestration of this kind of insecticides (Wilding et al., 2015). Our previous study showed that the transcripts of GSTs were able to be induced by the exposure to fenpropathrin (Liao et al., 2013), however, the link between GSTs and fenpropathrin resistance in *P. citri* is still not clear.

In this study, we compared the gene expression profiles between a fenpropathrin resistant population and a susceptible strain (SS) of *P. citri*. Overexpression of multiple genes, homologs of which have been associated with pyrethroids resistance in other pest arthropods, were found in resistant citrus red mites and the detoxification mechanisms of fenpropathrin in *P. citri* were discussed accordingly. Among those upregulated genes, one GSTs gene was annotated and determined as *PcGSTd1*, which has been characterized in our previous study (Liao et al., 2013). Subsequently, *in vitro* inhibition and metabolic assay of recombinant PcGSTd1 were designed to evaluate its potential metabolic activity toward fenpropathrin. Biochemical assay inferred a link between *PcGSTd1* and the oxidative effect caused by fenpropathrin. Additionally, an antioxidant role of *PcGSTd1* in the fenpropathrin detoxification was revealed through reverse genetic experiment as well. The present study provides some novel evidence of the function of GSTs gene and has the potential to be used as a biomarker in resistance monitoring and also in resistance management.

## Materials and methods

### Mites and chemicals

A laboratory strain of *P. citri* was collected in 2012 from the Banco orchard, and it was relatively susceptible to fenpropathrin based on bioassays and was reared without any exposure of acaricides, and considered as SS. This strain was maintained at 25 ± 1°C and 60% relative humidity (RH) under a 14:10 h light: dark condition. A fenpropathrin resistant strain (BB) was collected in 2014 from a citrus orchard in Beibei district of Chongqing, China, geographically close to the orchard of SS collection. The commercial formulation of fenpropathrin (20% EC) used for bioassay was purchased from Noposion (Noposion, Shenzhen, China). Analytical grade of fenpropathrin applied for HPLC (high performance liquid chromatograph) analysis was purchased from Sigma-Aldrich (St. Louis, MO, USA). The substrates for *in vitro* GSTs activity detection, 1-chloro-2,4-dinitrobenzene (CDNB), and reduced glutathione (GSH), and the inhibitor of GSTs, ethacrynic acid (ECA), were purchased from Sigma-Aldrich (St. Louis, MO, USA).

### Bioassays and fenpropathrin exposure

Fenpropathrin bioassay and exposure experiments were carried out using the leaf-dip method. Briefly, leaf disks with a diameter of 25 mm were made from fully expanded fresh lemon leaves and washed with deionized water before use. Twenty-five female adults were transferred onto a leaf disk with a soft brush, then the leaf disks with mites were dipped 5 s into serial dilutions of fenpropathrin. The test dilutions with serial concentration was diluted with deionized water using the commercial formulation of fenpropathrin. All the leaf disks with mites were subsequently incubated under climate-controlled conditions at 25 ± 0.5°C, 60% RH, and 14:10 h light: dark (L:D) photoperiod. Each dilution was tested in three replicates, including a water-dipped control. For the bioassays, mortality was calculated after 48 h. The surviving mites were collected at 24, 48, and 72 h after exposure of fenpropathrin with a sub-lethal concentration (LC_10_, 0.001 mg/L) using the same method as that of bioassay. The slope, 95% confidence intervals, median lethal concentration value (LC_50_) and LC_10_ were calculated by probit analysis using SPSS 16.0 (SPSS Inc., Chicago, IL, U.S.).

### RNA-seq library preparation and sequencing

Both the female adult mites from fenpropathrin resistant and SSs were collected for RNA-seq analysis. The total RNA was isolated using the RNeasy Plus Micro Kit (Qiagen GmbH, Hilden, Germany), and the genomic DNA was removed using a genomic DNA elimination column supplied with the kit. The RNA degradation and contamination was checked on 1% agarose gel, and the purity was confirmed further using the NanoPhotometer spectrophotometer (IMPLEN, Westlake Village, CA, USA). The quantification of RNA was measured using Qubit RNA Assay Kit in Qubit 2.0 Flurometer (Life Technologies, Carlsbad, CA, USA). RNA integrity was assessed using the RNA Nano 6000 Assay Kit of the Agilent Bioanalyzer 2100 system (Agilent Technologies, Santa Clara, CA, USA). A total amount of 3 μg RNA per sample was used for the RNA sample preparations. After cluster generation, the library preparation were sequenced on Illumina Hiseq 2500 platform and paired-end reads were generated. RNA-seq data were deposited to the NCBI Gene Expression Omnibus (GEO) under accession number SRP114745.

### RNA-seq data analyses

The raw reads were cleaned through removing adapter sequences, reads containing ambiguous bases, and low quality reads. The sequencing quality of the clean reads was evaluated based on the base-calling quality scores of Illumina's base-caller Bustard. Transcriptome assembly was accomplished using Trinity (Grabherr et al., 2011) with “min_kmer_cov” set to two by default and all other parameters as default set. Gene function was annotated based on the BLASTx against the NCBI non-redundant protein sequences (Nr), NCBI non-redundant nucleotide sequences (Nt), Protein family (Pfam), Clusters of Orthologous Groups of proteins (KOG/COG), Swiss-Prot, KEGG Ortholog database (KO), and Gene Ontology (GO) with a cutoff *E*-value of 10^−5^. To identity the differential expression genes between resistant and susceptible mites, the gene expression levels were estimated firstly by RNA-Seq by Expectation Maximization (RSEM) for each sample, and was further performed using the DEseq R package. *P*-value was adjusted using *q*-value. *Q*-value < 0.005 and |log2 (fold change)| > 1 was set as the threshold for significantly differential expressions.

### qRT-PCR

A total of 12 upregulated genes, which are potentially associated with pyrethroids resistance, were chosen to be validated by qRT-PCR method. The total RNA from two samples were reverse transcribed to synthesize the first-strand cDNA using GoScript Reverse Transcription System (Promega, Fitchburg, MA, USA). All the primers for qRT-PCR were designed in Primer 3.0 (http://primer3.ut.ee/) according to the partial sequence obtained by RNA-seq (Table S3). The qRT-PCR was performed on a Stratagene Mx3000P thermal cycler (Stratagene, La Jolla, CA, USA) with an initial denaturation at 95°C for 120 s, followed by 40 cycles of 95°C for 15 s, and 60°C for 30 s. The gene relative expression level was calculated according to the 2^−ΔΔCt^ or 2^−ΔCt^ method.

### Prokaryotic expression and purification of recombinant protein

The open reading frame (ORF) sequence of *PcGSTd1* with restriction site was amplified by high fidelity polymerase, PrimeSTAR Max Premix (Takara, Dalian, China), with the specific primers (Table S3). The 12.5 μL of PrimeSTAR Max Premix were used in the 25 μL of total volume PCR reaction with one cycle at 98°C for 2 min following with 34 cycles of 98°C for 10 s, 55°C for 20 s, and 72°C for 30 s, finally extending at 72°C for 5 min. The PCR products were purified using MiniBEST DNA Fragment Purification Kit (Takara, Dalian, China) and digested with a pair of restriction enzymes, *BamHI* and *NotI*, which were also used to digest the expression vector, pET-28a (Novagen, Madison, WI, U.S.). The resulting digests were subsequently constructed at 16°C for 30 min using a DNA ligation kit (Takara, Dalian, China), and the constructs were confirmed by DNA sequencing. The recombinant plasmids inserted with *PcGSTd1* were transformed to expression *Escherichia coli* TransBL21 (DE3; TransGen Biotech, Beijing, China). These were grown at 37°C on Luria-Bertani (LB) media containing 100 μg/mL kanamycin. Isopropyl 1-thio-b-D-galactoside (IPTG) was added to a final concentration of 1 mM to induce the production of recombinant proteins after the cell density reached at 0.6–1.0 at OD_600_.

The protocol for purification of recombinant protein of *PcGSTd1* mainly involved a protocol described previously (Qin et al., 2013). After further incubation for 4 h, cells from 1 L culture were harvested by centrifugation, and the resulting pellet was re-suspended in 90 mL 50 mM PBS buffer (pH 8.0) containing 0.5 M NaCl, 0.1% Triton X-100, and 0.05% Tween 20. The cell suspension was sonicated and centrifuged at 15,000 g at 4°C for 30 min. The supernatant (cleared lysate) was transferred to Ni-NTA spin column (Qiagen GmbH, Hilden, Germany) that were pre-equilibrated with above PBS. The Ni-NTA spin column was sequentially washed using 20 mL PBS buffer with a linear gradient of imidazole from 5 to 250 mM. There, recombinant GST was eluted with PBS containing 250 mM imidazole and dialyzed against TGE buffer (50 mM Tris, 0.5 mM EDTA, 50 mM NaCl, 5% glycerine, 1% glycine, pH 8.0). The purity of the recombinant GST was evaluated by 12% sodium dodecyl sulfate-polyacrylamide gel electrophoresis (SDS-PAGE).

### Activity assays of the recombinant PcGSTd1

Specific activity of GST was spectrophotometrically measured using a method described previously (Habig et al., 1974), with CDNB and GSH, in a total volume of 200 μL reaction mixture. The change of absorbance of CDNB conjugate was measured at 340 nm and 28°C for 5 min using xMark™ Microplate Spectrophotometer (Bio-Rad, Hercules, CA, U.S.). The protein content of PcGSTd1 was determined according to the method of Bradford using bovine serum albumin as a standard (Bradford, 1976). The values of *K*_m_ and *V*_max_ were determined using the Lineweaver-Burk plot using SigmaPlot 12.5 (Systat Software Inc., London, UK). For the inhibition assay of fenpropathrin against recombinant PcGSTd1, about 10 μg (5 μL) of GST protein was incubated with fenpropathrin (5 μL) at 28°C for 10 min, and then the mixture of GST and fenpropathrin (in 1–5% final concentration of methanol or acetone) was added to the reaction of 0.6 mM CDNB and 5 mM GSH in a total volume of 200 μL of 0.05 M Tris–HCl buffer (pH 7.5). The inhibitory effect was assayed using CDNB and GSH as substrates and ECA/fenpropathrin was used in a 0.05 mM final concentration. The IC_50_ for ECA was determined using ECA at various final concentrations (0.05, 0.1, 0.5, 1, and 2 mM). The reaction without the GST protein and the incubation of fenpropathrin were also measured in parallel.

### Metabolic assay

The assay of fenpropathrin metabolism was conducted at 30°C for 60 min with shaking at 1,200 rpm, in a total volume of 0.2 mL according to a method described previously (Riveron et al., 2014). The reaction was examined containing 0.05 M Tris-HCl buffer (pH 7.5), 2.5 mM GSH, 0.02 mg/mL fenpropathrin (the solvent was less 10% of the total reaction volume), and one unit of GST protein. Fenpropathrin and its metabolites were separated by the mobile phase of 80% A: 20% B (A: acetonitrile, B: 0.1% phosphoric acid in water) with a 1.4 mL/min of flow rate at 25°C. The change in absorbance at 210 nm was monitored and quantified by peak integration using Agilent 1260 LC (Agilent Technologies, CA, USA).

### RNAi through the leaf-mediated dsRNA feeding

The dsRNA of *PcGSTd1* was synthesized *in vitro* using Transcript Aid T7 High Yield Transcription Kit (Thermo Scientific, Waltham, MA, U.S.) according to the manufacturer's instructions. The resulting transcripts was purified to assure the quality of synthesized dsRNA. Additionally, the integrity of dsRNA was confirmed on a 1% agarose gel and its quantity was measured with a Nanovue UV–Vis spectrophotometer (GE Healthcare, Bucks, UK). On the basis of a method used for the gene silencing in whiteflies (Luan et al., 2013), a similar device was established to silence the gene of *P. citri* by dsRNA feeding through citrus leaf. Firstly, one tender citrus leaflet was detached from the citrus seedlings (*Aurantii fructus*) and washed with pure water. Then, the leaflet was incubated in oven at 60°C for 3–5 min and subsequently transferred into a 0.2 mL Axygen nuclease-free PCR tube containing 200 μL dsRNA or distilled water for a 1–2 h recovery period. After that, female adult mites from SS strain starved for 24 h previously, were transferred onto the leaf using a soft brush. The PCR tube with a citrus leaf were then transferred into a 50 ml plastic tube and covered with a piece of thin gauze tightly held with a rubber band. Finally, the devices were placed in an incubator under the condition of 25 ± 1°C, 50 ± 5% RH and 14:10 h light: dark photoperiod. The concentration of dsRNA was diluted to approximate 500 ng/μL. The solution in the PCR tube was supplied daily. After 48 h of incubation, 20 surviving female adults on the leaf were collected for total RNA isolation of each biological replicate. At 24 h after exposure to fenpropathrin with a sub-lethal concentration of LC_50_ (0.02 mg/L), mortality was calculated to investigate the susceptibility of ds*PcGSTd1*, ds*GFP* and nuclease-free water feeding mites. In the meantime, mites were collected for the total malondialdehyde (MDA) content detection. The mites were determined as dead if they cannot respond to the stimulation when stimulated with a soft brush.

### Measurement of lipid peroxidation level in *P. citri*

The lipid peroxidation level of mites after the exposure to fenpropathrin was investigated using Lipid Peroxidation MDA Assay Kit (Beyotime, Beijing, China) according to the manufacturer's instruction. After exposure, mites were homogenized in phosphate buffer (0.1 M, pH 7.5) on ice, and the homogenate was centrifuged at 15,000 g for 15 min. The pellets were discarded and the supernatant was filtered through Acrodisc 25 mm Syringe Filter (Pall Corporation, Port Washington, NY, USA). The filtered supernatant was used for the measurement of lipid peroxidation level, while corresponding protein content was measured according to the method of Bradford using bovine serum albumin as a standard (Bradford, 1976).

### Statistical analysis

Data were analyzed using SPSS v.16.0 (SPSS Inc., Chicago, IL, U.S.). All results here are showed as mean ± standard error. The differences of time-dependent responses to fenpropathrin exposure were analyzed using one-way analysis of variance (ANOVA). For the RNAi experiment, the significant differences of *PcGSTd1* expression, MDA content and mortality after fenpropathrin exposure were also analyzed using one-way ANOVA. The level of significance of the means was then separated by Fisher's LSD multiple comparison test (*P* < 0.05).

## Results

### RNA-seq analyses of fenpropathrin-resistant and susceptible mites

The bioassay results showed that the citrus red mite from Beibei citrus orchard of Chongqing, China was highly resistant to fenpropathrin compared to the susceptible (SS) strain. The median lethal concentration (LC_50_) of fenpropathrin against BB population was 230-fold relative to that of SS strain (Table 1).

**Table 1:** Resistance levels of fenpropathrin resistant population (BB) compared to susceptible strain (SS) of *P. citri* as determined by bioassay.

**Strains**	***n***	**Slope**	**χ^2^**	**LC_50_ (95% CL), mg·L^−1^**	**Resistance ratio**
BB	566	2.98	5.7	5.30 (4.84–5.79)	230
SS	310	0.51	1.6	0.02 (0.01–0.06)	–

*n, tested numbers of mites; χ^2^, goodness-of-fit test; LC_50_, median lethal concentration; CL, confidence limits*.

Two paired-end RNA-seq libraries were generated for pools of each BB and SS mites and sequenced giving 61,746,562 and 56,088,058 clean reads for the resistant and susceptible samples, respectively. To identify the differentially expressed genes between BB and SS samples, the differences of gene expression level were comparatively analyzed using FPKM estimation method. A total of 861 transcripts were found significantly differentially accumulated between BB and SS mites. Among those significantly regulated transcripts in the two samples, 499 were upregulated and 362 were downregulated in the fenpropathrin-resistant samples compared with the SS (Figure S1). Further function annotation results showed that, those upregulated transcripts include GSTs, CarEs, cytochrome P450s (P450s), cuticular proteins, heat shock proteins (Hsps), ATP-binding cassette transporters (ABC transporters), and antioxidant enzymes (oxidases/peroxidase and thioredoxin peroxidase) (Table 2). Amongst those upregulated metabolic detoxification enzymes, only one transcript (c24614_g3) encoding for GST was found significantly upregulated with a fold change of 2.3 and this gene has been identified as *PcGSTd1* in our preliminary work (Table 2). Additionally, it is worthy noted that both of the two genes encoding antioxidant enzymes, oxidases/peroxidase, and thioredoxin peroxidase, were also significantly upregulated in fenpropathrin resistant mites with a fold change of 28.9 and 27.0, respectively.

**Table 2:** Partial upregulated genes in fenpropathrin resistant population of *Panonychus citri* analyzed by DGE.

**Gene families**	**Gene ID**	**Fold-change**	**Log_2_ fold-change**	**Q-value**	**Orthologs in *Tetranychus urticae***	**Description**
Glutathione *S*-transferases	c24614_g3	2.3	1.2	2.38E-06	tetur03g07920	Glutathione *S*-transferase, delta class TuGSTd06
Cytochrome P450s	c14555_g1	2.5	1.3	1.33E-06	tetur26g01470	Cytochrome P450 CYP385C1
	c26785_g1	2.1	1.1	3.01E-07	tetur25g02050	Cytochrome P450 CYP389A1
Carboxylesterases	c27234_g1	3.4	1.8	2.61E-17	tetur37g00340	Carboxyl/cholinesterase TuCCE68
	c14529_g1	2.3	1.2	1.67E-258	tetur20g03250	Carboxyl/cholinesterase TuCCE50
	C28241_g1	3.3	1.7	2.1964E-29	Tetur16g02380	Carboxyl/cholinesterase TuCCE40
Cuticle proteins	c24506_g1	53.6	5.7	1.33E-06	tetur23g01280	Cuticular protein analogous to peritrophins 1-A CPAP 5
	c25233_g1	17.1	4.1	0.0041417	tetur05g04610	Cuticular protein analogous to peritrophins 1-A CPAP 11
	c18540_g1	5.2	2.4	0.002223	tetur32g02090	Cuticle protein CPR 2
Heat shock proteins	c27361_g1	69.5	6.1	1.92E-08	tetur11g01700	Heat shock protein Hsp70
	c24757_g3	40.8	5.4	4.49E-05	tetur07g03840	Heat shock protein 90
Transporters	c27202_g1	30.3	4.9	0.00080948	tetur01g05940	ABC-transporter, class H
	c25885_g1	3.4	1.7	0.00044308	Tetur19g01160	ABC-transporter, class G
	c28777_g1	2.7	1.4	1.02E-05	tetur01g10390	ABC-transporter, class C
Antioxidant enzymes	c27875_g1	28.9	4.9	0.0012241	tetur03g07740	Oxidase/Peroxidase
	c20599_g1	27.0	4.8	0.0020579	tetur08g00400	Thioredoxin peroxidase

### Validation of the upregulated genes in resistant mites

**Figure 1:**
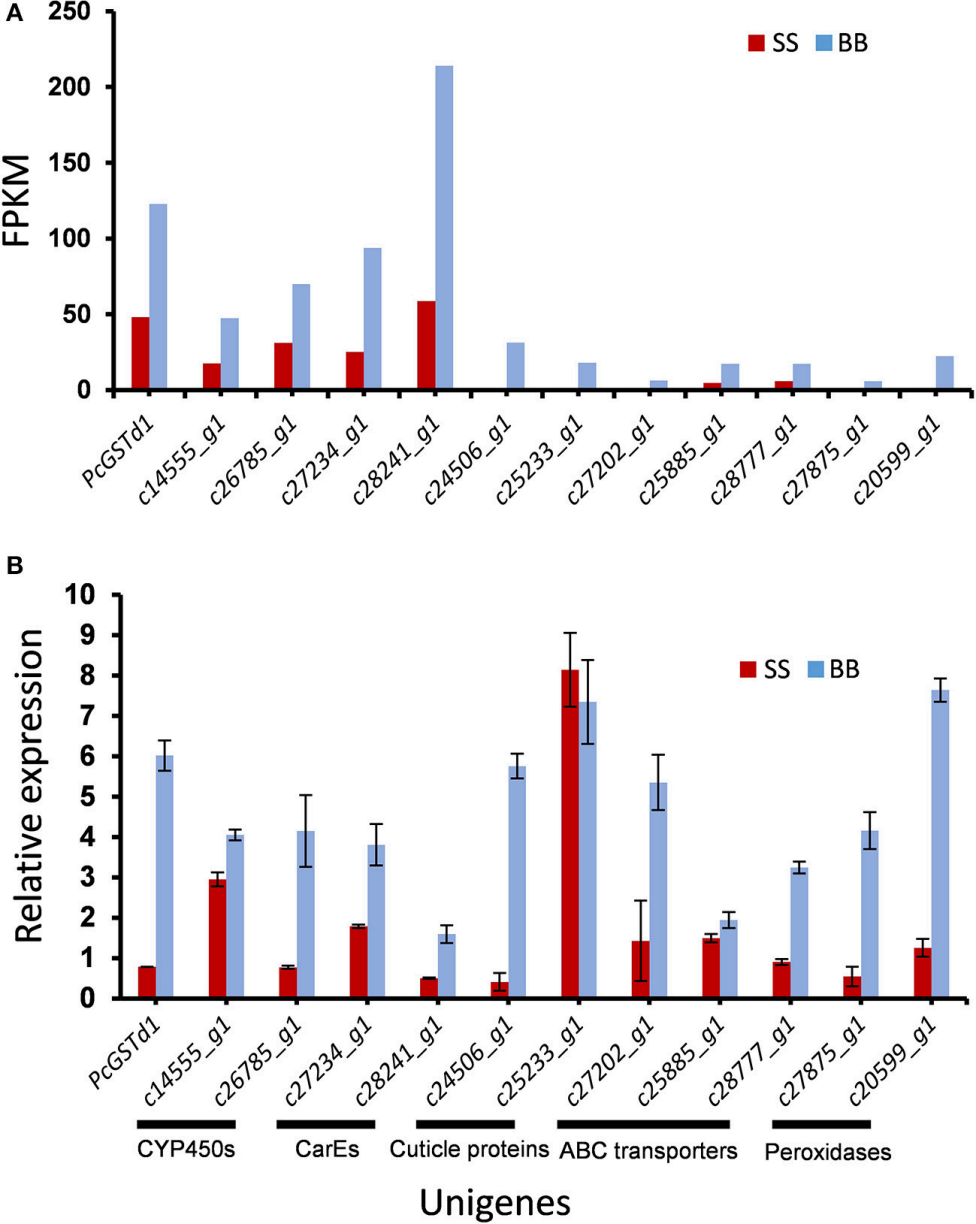
Significantly upregulated transcripts potentially associated with fenpropathrin resistance in citrus red mite. **(A)** Quantification level in FPKM (Fragment Per Kilobase of exon model per Million mapped fragment) between fenpropathrin resistant and susceptible mites, gene expression was quantified by FPKM to minimize the influence of variation in gene length and total number of reads; **(B)** qRT-PCR verification of higher accumulated transcripts in resistant mite.

Based on their potential relevance with pyrethroids resistance, a total of 12 genes upregulated in fenpropathrin-resistant mites were validated using with quantitative real time-PCR (qRT-PCR). Comparatively, the data analyzed by qRT-PCR was almost identical with RNA-seq data (Figure 1A). The direction of fold changes were conserved for 11 of the tested 12 transcripts (Figure 1B). In addition that, the fold change of relative expression level for *PcGSTd1* detected by qRT-PCR (8-fold) was higher than the data obtained from RNA-seq (Figure 1B).

### Upregulation of *PcGSTd1* after the exposure of fenpropathrin

**Figure 2:**
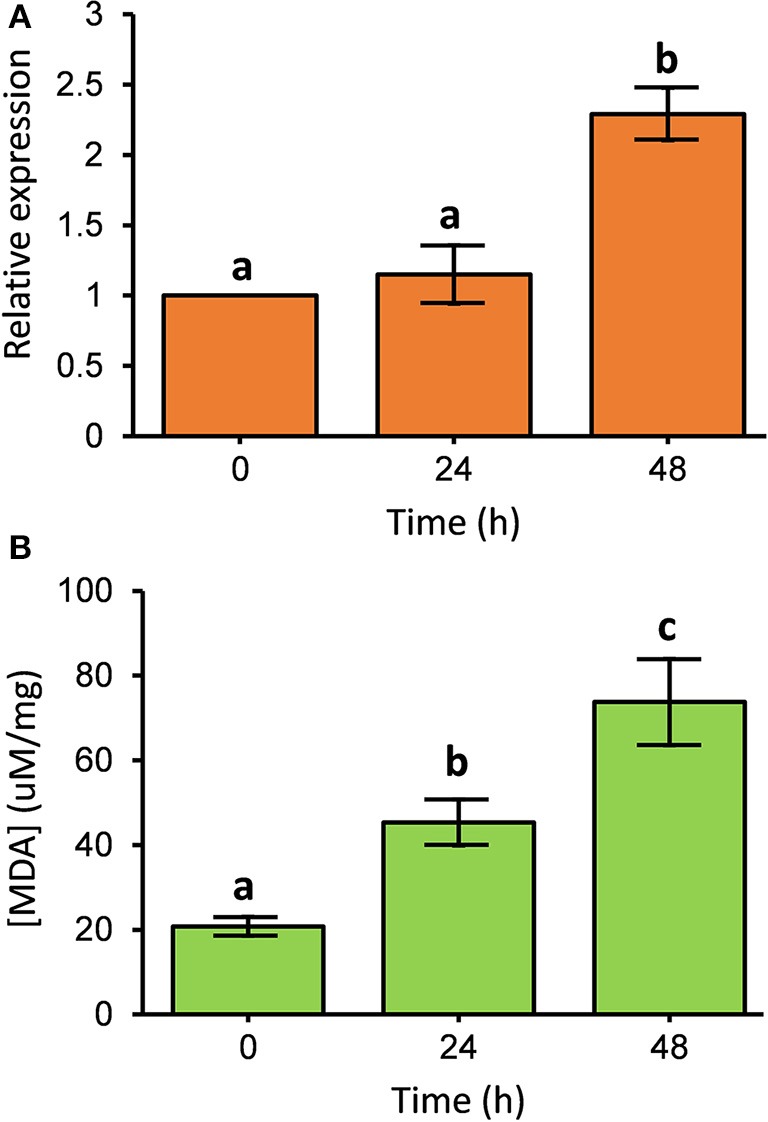
Expression pattern of *PcGSTd1* and lipid peroxidation level after fenpropathrin exposure. **(A)** Expression pattern of *PcGSTd1* response to the exposure of fenpropathrin. The mRNA level in each treatment group is shown as a fold change relative to the mean expression in the control (0 h), which was defined as a basal value of 1. **(B)** Total MDA content after fenpropathrin treatment. Different letters on the standard deviation bars indicate significant differences in expression of *PcGSTd1* and total MDA content between each time-point based on the Fisher's LSD multiple comparison test (*P* < 0.05).

To investigate the transcripts of *PcGSTd1* in response to fenpropathrin exposure, the time-dependent relative expression of *PcGSTd1* to *GAPDH* was quantified by qRT-PCR. Interestingly, after 48 h of exposure to a sub-lethal concentration of fenpropathrin, the mRNA expression level of *PcGSTd1* was more than 2.3 times higher than that of untreated control (*P* < 0.05; Figure 2A). This result indicate that the expression of *PcGSTd1* could be induced by fenpropathrin at a sub-lethal concentration. The inductivity of *PcGSTd1* response to fenpropathrin was consistent with its overexpression in fenpropathrin resistant mites as well. Therefore, to elucidate the potential association between GSTs superfamily gene and resistance to fenpropathrin, *PcGSTd1* would be the primary target GST gene in *P. citri* for further analysis.

### Increased lipid peroxidation level after the exposure of fenpropathrin

The content of MDA was measured to determine whether the lipid peroxidation level changes after the exposure of fenpropathrin. As expected, we found that the MDA content in *P. citri* was gradually increased within 48 h after fenpropathrin treatment. The concentration of MDA in *P. citri* at 24 and 48 h post of treatment with sub-lethal concentration of fenpropathrin were 45.3 and 73.7 μM/mg, respectively. Both of these were significantly higher than that measured in control (20.8 μM/mg) (Figure 2B). Thus, the data described above suggest that fenpropathrin exposure resulted in the oxidative stress and increased the lipid peroxidation level in *P. citri*.

### *In vitro* metabolic and inhibition assays with recombinant *PcGSTd1* protein

**Figure 3:**
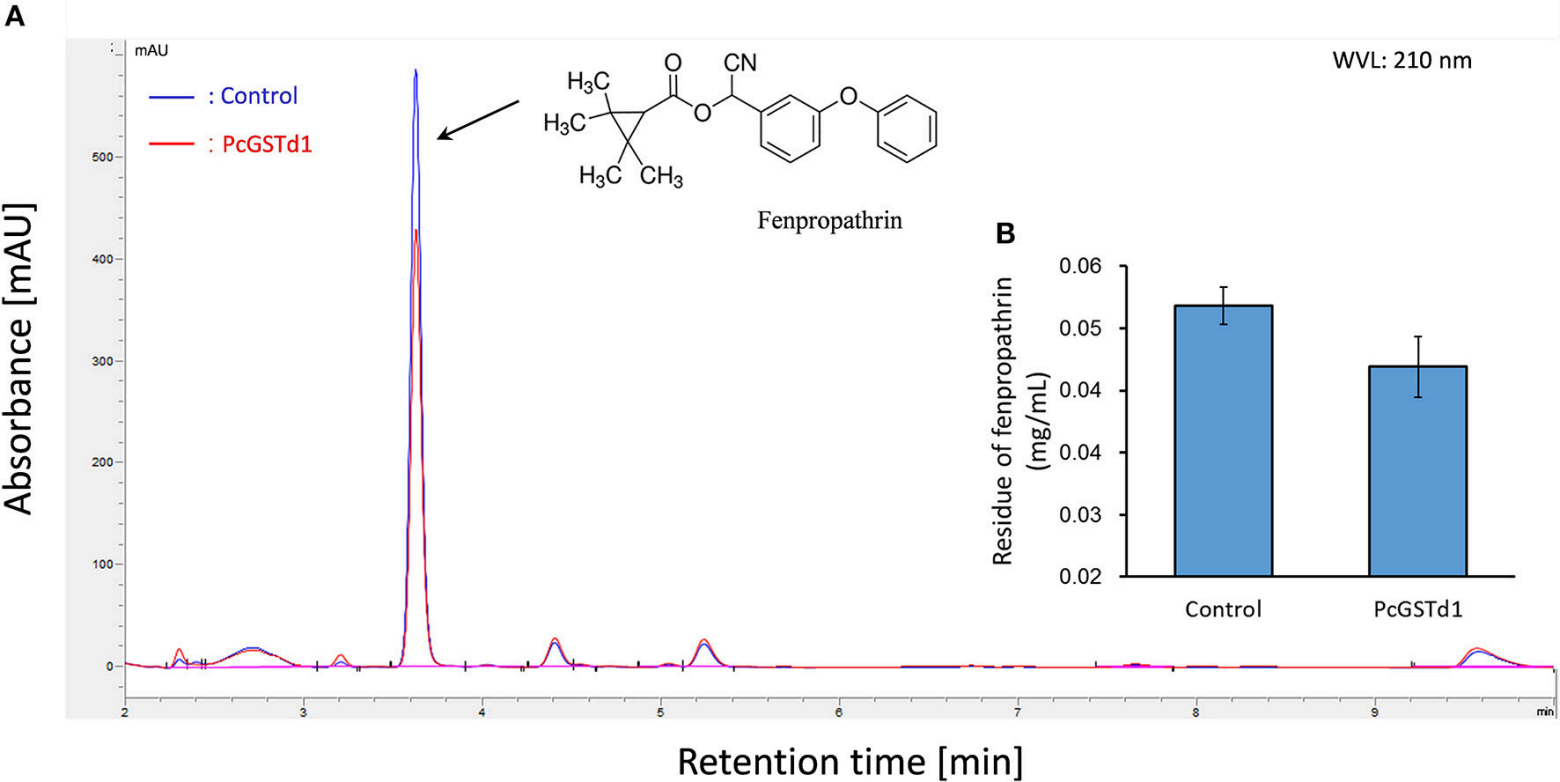
Metabolic assays of fenpropathrin by PcGSTd1. **(A)** Chromatography of fenpropathrin. The blue line refers to incubation of fenpropathrin and GSH with the boiled (inactivation) recombinant PcGSTd1 protein. The red line refers to incubation of fenpropathrin and GSH with active recombinant PcGSTd1 protein. **(B)** Comparison of fenpropathrin residue between the incubation of fenpropathrin with inactive and active PcGSTd1 protein. Both incubation with active and inactive PcGSTd1 were replicated for 6 times.

To detect whether PcGSTd1 can directly metabolize fenpropathrin, the recombinant protein of *PcGSTd1* was produced in *E. coli* and further purified abundantly. The catalytic activity of recombinant enzyme was investigated using the common substrates for GSTs, CDNB, and GSH, and its kinetic parameters and enzymatic characteristics were also analyzed (Table S1). The results showed that the purified recombinant PcGSTd1 possess a relative higher activity to CDNB. However, after incubation with recombinant PcGSTd1, the quantity of fenpropathrin have no significant changes compared with control (Figure 3). Meanwhile, almost no new signal peak which may represent the potential metabolites was detected in the reaction of active recombinant PcGSTd1 and fenpropathrin (Figure 3). Similarly, the activity of PcGSTd1 toward CDNB kept stable when added with fenpropathrin, which suggesting fenpropathrin was unable to compete with CDNB, as a potential substrate for PcGSTd1, under the condition established in this study. However, it was determined that the activity of PcGSTd1 could be inhibited by ECA under the same condition and the median inhibitory concentration was calculated accordingly (Table S2).

### RNAi of *PcGSTd1* to exposure of fenpropathrin

**Figure 4:**
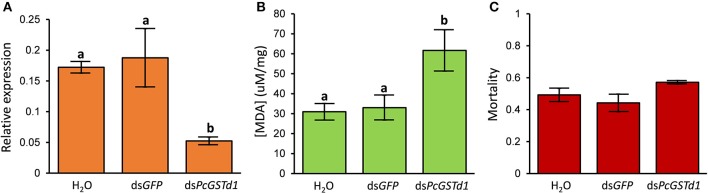
RNAi of *PcGSTd1* and investigation of lipid peroxidation level and susceptibility to fenpropathrin. **(A)** Changes in the mRNA level of *PcGSTd1*. The RNAi efficiency was investigated 48 h after the gene silencing. **(B)** MDA content of nuclease-free water, ds*GFP* and ds*PcGSTd1* feeding mites after the exposure of fenpropathrin. **(C)** Mortality of control (water and ds*GFP*) and ds*PcGSTd1*-silencing mites after the exposure of fenpropathrin. Fenpropathrin bioassay was conducted 24 h after the gene silencing treatment. Results were mean and standard errors of three biological replications (*n* = 3). Different letters on the standard deviation bars indicate significant differences between each group based on the Fisher's LSD multiple comparison test (*P* < 0.05).

Combined with the increased mRNA expression of *PcGSTd1* and lipid peroxidation level after the exposure of fenpropathrin, we supposed that *PcGSTd1* may play an important role in the modulation of lipid peroxidation induced by fenpropathrin and indirectly involved in the detoxification of fenpropathrin. Thus, to verify the possible detoxification function conferred by *PcGSTd1*, RNAi experiments for *PcGSTd1* were conducted using the leaf-mediated dsRNA feeding method. Results show that the relative expression of *PcGSTd1* was significantly decreased after the RNAi treatment. Compared with the treatment with ds*GFP*, the mRNA expression of *PcGSTd1* was significantly silenced with about 70% of RNAi efficiency after the continuous supplying of ds*PcGSTd1* for 48 h (Figure 4A). It is interesting that the MDA content in ds*PcGSTd1*-feeding mites were significantly higher than that in those mites feeding with ds*GFP* or water after the exposure of fenpropathrin. The concentration of MDA in ds*PcGSTd1*-feeding mites, ds*GFP*-feeding mites and water-feeding mites were 61.6 ± 10.36, 33.0 ± 6.22, and 30.9 ± 4.15 μM/mg, respectively (Figure 4B). These results suggested that *PcGSTd1* might participate in the regulation of MDA content in *P. citri*, thus weaken the lipid peroxidation induced by fenpropathrin. However, after the treatment with sub-lethal concentration of fenpropathrin, no significant change was observed between the mortalities of ds*PcGSTd1*-feeding mites and control, even though the former was a little higher than the latter (Figure 4C).

## Discussion

As one membership of the largest chemical subfamily of pyrethroids, fenpropathrin exhibit a greater range of insecticidal/acaricidal activity and enhanced overall insecticidal potency compared to earlier synthetic pyrethroids. The earlier investigation has revealed that the metabolic pathway of pyrethroids degradation initially constitute of the oxidative, reductive, and hydrolytic processes, which need the participation of necessary catalytic enzymes including P450 monooxygenases, esterases and GSTs (Soderlund et al., 2002). As we expected, RNA-seq analysis in this study showed that the transcripts of two P450s, three CarEs, and one GSTs were upregulated in fenpropathrin resistant population of *P. citri* compared to the SS. The increased expression at RNA level of the three major detoxification enzymes (P450s, CarEs, and GSTs) detected in pyrethroids resistant *P. citri* are consistent with previous studies conducted among various species including insects and mites (Muller et al., 2007; Dermauw et al., 2013; Zhu et al., 2014; Cong et al., 2016). Alignment analysis of amino acid sequences with the analogous from *T. urticae* showed that two P450 proteins (c14555 and c26785) are belonged to CYP385 and CYP389 subfamily, respectively. Interestingly, it is reported recently that one P450 gene from CYP389 subfamily in *T. cinnabarinus* was confirmed to contribute to fenpropathrin resistance collaboratively with other five P450s genes (Shi et al., 2016b). Although the precise function of c26785 in *P. citri* remains further investigation, both of our results and functional analysis of P450 genes in *T. cinnabarinus* demonstrate the P450s in CYP389 subfamily might play an important role in the fenpropathrin detoxification process. Additionally, two upregulated CarEs transcripts (c14529 and c27234) in fenpropathrin resistant mites were identified as *PcE1* and *PcE9*, respectively, which has been characterized in our preliminary study and proved conferring resistance to fenpropathrin (Shen et al., 2016). Thus, our current evidences are sufficient to support that fenpropathrin resistance in *P. citri* be partially attributed to the overexpression of *PcE1* and *PcE9*.

Recognized as one of the most important secondary metabolic detoxification enzymes, GSTs also play an important role in conferring resistance to pyrethroids. Elevated transcripts of GSTs gene, which supposed to enhance the capacity to detoxify, have been investigated in various pyrethroids resistant pest insects and mites (Mounsey et al., 2010; Lumjuan et al., 2011; Dermauw et al., 2013; Cong et al., 2016). Similarly, among the differential accumulated genes here, one GSTs gene (*PcGSTd1*) was detected to be upregulated in fenpropathrin resistant mites. The qRT-PCR results confirmed that *PcGSTd1* transcripts was also upregulated after the exposure of sub-lethal concentration of fenpropathrin. In this context, we thereby predicted that *PcGSTd1* might be involved in the detoxification of fenpropathrin in *P. citri*. So far, little to no study provides the evidence showing pyrethroids degradation could be catalyzed by GSTs. It was reported that GSTe2 from wild resistant population of mosquitoes can metabolize permethrin directly, even though its definite metabolites remain unknown (Riveron et al., 2014). Applying similar methods, however, we have not collected empirical evidences in this study inferring PcGSTd1 can metabolize or sequestrate fenpropathrin directly. It is speculated that *PcGSTd1* may participate in the detoxification of fenpropathrin via additional way such as protecting them against the peroxidation production induced by fenpropathrin like as occurred in *N. lugens* (Vontas et al., 2001).

MDA has been known as one of the lipid peroxidation products and its content reflect the ROS level at some extend. Thus, MDA has been used as the biological marker of oxidative stress (Del Rio et al., 2005). Intriguingly, time-dependent gradually elevated MDA was investigated after the exposure of fenpropathrin (Figure 2B), indicating fenpropathrin indeed elicit oxidative stress in the citrus red mite. These results may provide a corresponding explanation why antioxidant enzymes were accumulated much more in fenpropathrin resistant mites, when compared with the SS. Accordingly, the significant upregulation of two peroxidases in fenpropathrin resistant mite suggests that constitutive high level expression of antioxidant enzymes might be contributed to its survival in the exposure of fenpropathrin. This observation was also supported by previous studies in which the increased transcription of superoxide dismutase (SOD) gene, except peroxidases, was detected in pyrethroids resistant mosquitos (Muller et al., 2007, 2008). In addition to peroxidases, GSTs have also been implicated in protecting organisms through alleviating lipid peroxidative damage (Singh et al., 2001; Vontas et al., 2001). Thus, we speculated that upregulation of *PcGSTd1* in resistant population and after the exposure of sub-lethal fenpropathrin might contribute to reducing the lipid peroxidation and protecting mites from the oxidative damage caused by fenpropathrin.

Finally, to confirm the hypothesis that *PcGSTd1* play an antioxidant role in fenpropathrin detoxification, we compared the lipid peroxidation level after exposure of sub-lethal concentration of fenpropathrin in *PcGSTd1*-silencing mites with that in control. As we expected, more MDA were represented in *PcGSTd1*-silencing mites after fenpropathrin treatment compared with control. Despite the direct evidence showing activity of *PcGSTd1* to degrade MDA remains collected, differences caused by RNAi of *PcGSTd1* strongly indicate that this GST, for certain, participates in the elimination of lipid peroxidation products induced by fenpropathrin. However, no significant change of susceptibility to fenpropathrin has been investigated between RNAi treatment and control. These results suggest that, on the one hand, change on MDA content caused by RNAi of *PcGSTd1* in this study may not be sufficient to result in the susceptibility alteration of *P. citri* to fenpropathrin, despite the expression of *PcGSTd1* has been efficiently suppressed. Furthermore, the primary knockdown/killing mechanism of fenpropathrin, as all the other pyrethroids, should be the neurotoxicity through inactivating sodium channel (Soderlund et al., 2002), although the oxidative stress to *P. citri* also exist objectively. Our results, in some sense, indicate the oxidative damage caused by fenpropathrin likely be merely regarded as a minor cause rather than the key factor result in the toxicity to *P. citri*. Even so, we cannot ignore readily the antioxidant role of *PcGSTd1* in fenpropathrin detoxification/resistance. In the future study, more efficient reverse genetic tool, such as CRISPR/Cas9 system, is promising to be applied for uncovering the role of *PcGSTd1* in fenpropathrin resistance.

## Conclusions

Our study provides the transcriptional evidences hinting at a multiple metabolic mechanism involved in the development of fenpropathrin resistance in the field population of *P. citri*. In order to understand comprehensively the underlying mechanisms conferring fenpropathrin resistance, those upregulated resistance-associated transcripts identified in resistant mites would be considered as the key candidate target genes for further function analysis. Alternatively, GSTs play an antioxidant role in alleviating the oxidative stress caused by fenpropathrin. In conclusion, the whole results obtained in this study extend our knowledge about the potential molecular mechanisms of pyrethroid resistance in *P. citri* and demonstrate the detailed role of GSTs functioning as antioxidant enzymes in detoxification of pyrethroid.

## Author contributions

C-YL, WD, and J-JW: Designed research; C-YL: Performed all of the experiments with the help of Y-CF, GL, and X-MS; J-JW: Provided the materials; C-YL and S-HL: Analyzed data; C-YL, WD, and J-JW: Wrote the paper.

### Conflict of interest statement

The authors declare that the research was conducted in the absence of any commercial or financial relationships that could be construed as a potential conflict of interest.
