# Supplementary material for: Antioxidant Role of PcGSTd1 in Fenpropathrin Resistant Population of the Citrus Red Mite, Panonychus citri (McGregor)
Source: Front Physiol. 2018 Mar 29;9:314. doi: 10.3389/fphys.2018.00314 (PMC5884870; doi:10.3389/fphys.2018.00314)
Supplement: Supplementary file 1 [file Presentation1.pdf]

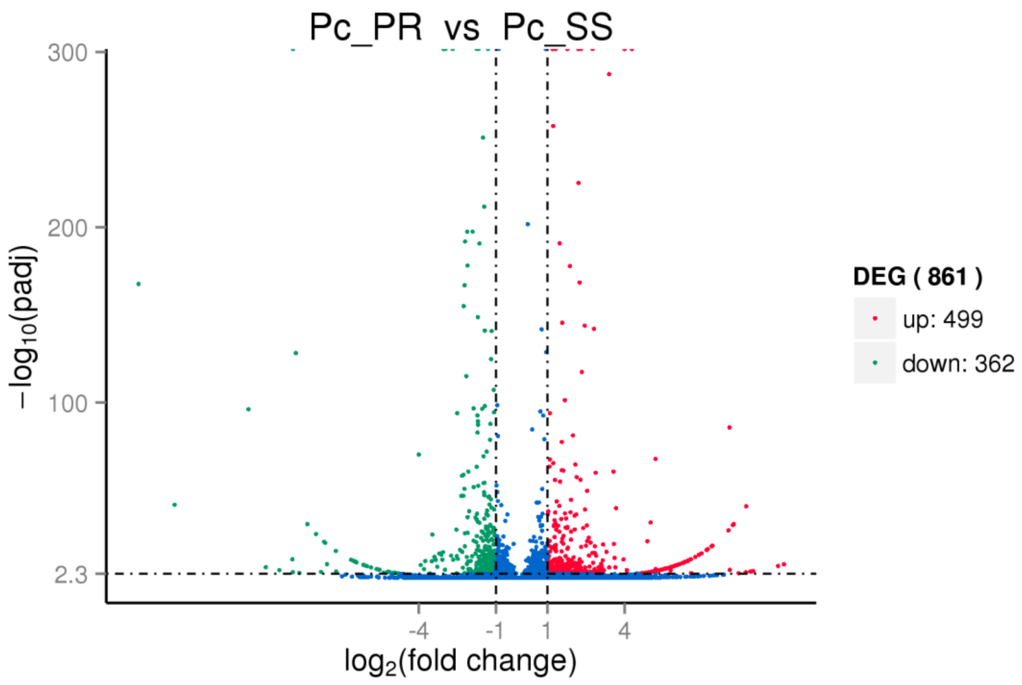

Figure S1. Volcano plot showing the differential expression pattern between fenpropathrin-resistant mites (PR) and the susceptible strain (SS), with twofold-change cutoff and *Q* value < 0.005.

Table S1. Specific activity and kinetic parameters of PcGSTd1 towards the conjugate of CDNB and GSH.

	Specific activity (μmol/min/mg)	K_m (mM)	V_{max} (μmol/min/mg)
CDNB	7.83 ±0.32	1.82 ±0.49	15.77 ±3.78

All values are means ± S.E. of three independent experiments. Results were determined by varying the concentrations of CDNB (0.0125-0.3 mM) and at fixed concentration of GSH (5 mM).

Table S2. Inhibitory effect on the activity of PcGSTd1 towards CDNB.

	IC_{50} (mM)
Ethacrynic acid	1.13 ±0.12
Fenpropathrin	n.d.

All values are means ± S.E. of three independent experiments. The inhibitory effect was assayed using CDNB and GSH as substrates and ECA/fenpropathrin was used in a 0.05 mM final concentration. The IC_{50} for ECA was determined using ECA at various final concentrations (0.05, 0.1, 0.5, 1 and 2 mM). N.d.: not detected.

Table S3. List of primers used in this study.

**Primer names**	**Oligonucleotide sequences**	**Description**
PcGSTd1-q-F	GAGTCGCTCGAGTTTTGGTC	qRT-PCR
PcGSTd1-q-R	GAATTACCTGGGGCGAGTTT	
c14555-q-F	GGTGTCTACGATGGCCTACA	qRT-PCR
c14555-q-R	AGGTGTCAACAGGGATCGTA	
c26785-q-F	CAGTCTGATAAGTCGAGCGC	qRT-PCR
c26785-q-R	GTGTCAAGTTGAGAGCAGTGG	
c27234-q-F	ATTCCTTACGCTGAGCCACCA	qRT-PCR
c27234-q-R	TCCTCGCTGAAAGAAACACCAC	
c28241-q-F	GGCTGAAGACACGGATGAAGAT	qRT-PCR
c28241-q-R	TCCACCCTGAAAACCACCAC	
c24506-q-F	GTCAAACAAGTCGCAGCTCA	qRT-PCR
c24506-q-R	AGACTTTGCATCCATTGGCG	
c25233-q-F	AGTTCCCAACCAATTCAACAGA	qRT-PCR
c25233-q-R	GGTTTCCTTCAATGGTCCACC	
c27202-q-F	TTCTCGACGAGCCTACAGTC	qRT-PCR
c27202-q-R	CAGTGTGAGAAGTCTTGCCG	
c25885-q-F	TGGTACTCTTGTGCGTCCTT	qRT-PCR
c25885-q-R	GCGCACCAAATAGTAGACCG	
c28777-q-F	GGTCGCTGAGAGAAGATCGA	qRT-PCR
c28777-q-R	ATCCGACGGCTCTGAAGTAG	
c27875-q-F	TGGTATCGTGGGACCAACAT	qRT-PCR
c27875-q-R	GCGGCTAATGTTGCAGTCTT	
c20599-q-F	GGTGGTCTCGGTGGTCTTAA	qRT-PCR
c20599-q-R	ACGGGAAGATCATTGACGGT	
GAPDH-q-F	CTTTGGCCAAGGTCATCAAT	qRT-PCR
GAPDH-q-R	CGGTAGCGGCAGGTATAATG	
ds*PcGSTd1*-F	T7-CCGAATTCACCTCAATCAAC	dsRNA synthesis
ds*PcGSTd1*-R	T7-AAGTGTCGGCCATTAGGT	
ds*GFP*-F	T7-GTGAAGGTGATGCAACATACG	dsRNA synthesis
ds*GFP*-R	T7-CCATCGCCAATTGGAGTAT	
PcGSTd1-28a-F	*CG*GGATCCATGTCCATCCAATTGTTTC	*BamHI*
PcGSTd1-28a-R	*ATTT*GCGGCCGCTTATTTTGAAGCTTTGGAT	*NotI*

T7 promoter sequence: TAATACGACTCACTATAGGG
